# A modular and compact portable mini-endstation for high-precision, high-speed fixed target serial crystallography at FEL and synchrotron sources

**DOI:** 10.1107/S1600577515016938

**Published:** 2015-10-06

**Authors:** Darren A. Sherrell, Andrew J. Foster, Lee Hudson, Brian Nutter, James O’Hea, Silke Nelson, Olivier Paré-Labrosse, Saeed Oghbaey, R. J. Dwayne Miller, Robin L. Owen

**Affiliations:** a Diamond Light Source, Harwell Science and Innovation Campus, Didcot, Oxfordshire OX11 ODE, UK; b LCLS, SLAC National Laboratory, Menlo Park, CA 94025, USA; c Department of Chemistry and Physics, University of Toronto, 80 St George St, Toronto, ON M5S 1A8, Canada; d The Max Planck Institute for the Structure and Dynamics of Matter, and Hamburg Centre for Ultrafast Imaging, CFEL Building 99, Luruper Chaussee 149, Hamburg 22761, Germany

**Keywords:** fixed-target serial femtosecond crystallography, portable endstation, motion control, sample delivery

## Abstract

The design and implementation of a compact and portable sample alignment system suitable for use at both synchrotron and free-electron laser (FEL) sources and its performance are described. The system provides the ability to quickly and reliably deliver large numbers of samples using the minimum amount of sample possible, through positioning of fixed target arrays into the X-ray beam. The combination of high-precision stages, high-quality sample viewing, a fast controller and a software layer overcome many of the challenges associated with sample alignment. A straightforward interface that minimizes setup and sample changeover time as well as simplifying communication with the stages during the experiment is also described, together with an intuitive naming convention for defining, tracking and locating sample positions. The setup allows the precise delivery of samples in predefined locations to a specific position in space and time, reliably and simply.

## Abstract 2

A portable sample viewing and alignment system is described which provides fast and reliable motion positioning for fixed target arrays at synchrotrons and free-electron laser sources.

## 1. Introduction   

Until recently almost all macromolecular crystal structures were determined from either a single or a small number of crystals. While multi-crystal data collection has been normalized in some fields, for example, room-temperature crystallography or micro-crystallography, even in these cases the number of crystals used for structure solution usually remains relatively small. Typical examples of the approximate number of crystals used are: 47 crystals for the A22 wild type and 77 crystals for the A22 VP2 H93C type foot and mouth disease virus structures determined at room temperature (Porta *et al.*, 2013 ▸), and 30 crystals used in the structure determination of the class B GPCR corticotrophin (Hollenstein *et al.*, 2013 ▸). In contrast, free-electron lasers have driven the emergence of serial femtosecond crystallography (SFX) where a structure is determined from many tens or even hundreds of thousands of crystals (Chapman *et al.*, 2011 ▸). The dramatic increase in the number of crystals required is driven by the duration and intensity of X-ray free-electron laser (XFEL) pulses: with ∼10^12^ photons delivered in 5–50 fs only a single still image from each crystal can be collected before destruction.

A number of sample delivery methods have been developed to facilitate SFX and crystallographic data collection at XFELs, many of which are summarized by Chavas *et al.* (2015 ▸). The most widely used has been the liquid jet or gas dynamic virtual nozzle (DePonte *et al.*, 2009 ▸), where a stream of crystal-containing liquid flows through the beam path. This approach provides a liquid jet of similar diameter to the size of the crystals. Approaches that greatly reduce the level of sample consumption of liquid jets such as the use of lipidic cubic phase (Weierstall *et al.*, 2014 ▸) and nanoflow electrospinning (Sierra *et al.*, 2012 ▸) have also been developed.

Goniometer-based approaches, more akin to traditional data collection methods at synchrotrons, have also been used at XFELs (Cohen *et al.*, 2014 ▸; Hirata *et al.*, 2014 ▸) allowing data collection from a number of positions and/or angles on a single crystal. The successful use of synchrotron instrumentation at XFELs has also been reflected through the emergence of serial crystallography at synchrotrons (Gati *et al.*, 2014 ▸; Coquelle *et al.*, 2015 ▸; Botha *et al.*, 2015 ▸).

Fixed target approaches with crystals mounted on a silicon wafer described by Hunter *et al.* (2014 ▸), where rigidly mounted crystals are rastered through the beam, are more closely related to goniometer-based approaches. The development of microfluidic flow cells (Heymann *et al.*, 2014 ▸; Lyubimov *et al.*, 2015 ▸) or grid-based wafers (Zarrine-Afsar *et al.*, 2012 ▸; Roedig *et al.*, 2015 ▸; Mueller *et al.*, 2015 ▸; Feld *et al.*, 2015 ▸) holding crystals in evenly spaced, well characterized positions offers the prospect of very high hit rates and structure solution from very small sample volumes. This latter feature is important for precious protein samples as the loading procedure uses the least amount of protein possible to attain large numbers of crystals for SFX. Some designs allow all protein not successfully loaded onto the chip to be recovered in the eluent of the loading process (Mueller *et al.*, 2015 ▸). It also becomes possible to collect multiple images from a crystal, which is relevant, for example, at FELs if the X-ray beam is attenuated or monochromatic.

The instrumentation described here is intended to facilitate fixed target SFX (FT-SFX) at both synchrotron and FEL sources. It is a compact and portable setup, which satisfies the need for fast, precise and reproducible positioning of samples and can be easily integrated into different beamline environments including vacuum.

## 2. Instrumentation   

**Figure 1:**
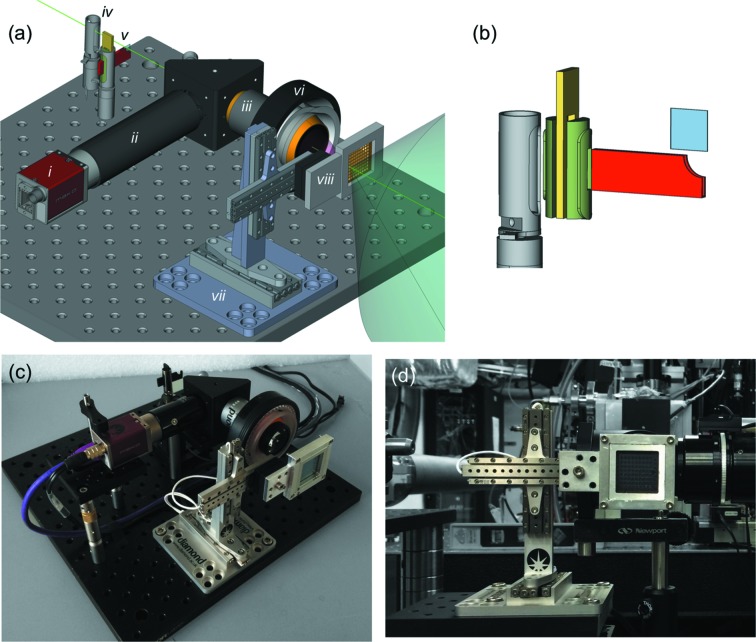
(*a*) CAD of the setup showing a typical arrangement. Components are labeled as (i) CCD camera, (ii) lens tube, (iii) drilled objective, (iv) rotary shutter, (v) rotary shutter with filter holder insert, (vi) ring light, (vii) stages and (viii) sample holder. Supporting elements have been removed for clarity. (*b*) Close-up of insert to convert rotary shutter into a filter holder. Photographs of (*c*) setup offline and (*d*) at XPP, LCLS.

The instrument consists of several modular components to maximize flexibility and these can be used together as described, in subsets or individually. The complete system allows simple control and motion of samples mounted on a high-speed, high-precision *xyz* stage with synchronized attenuation and shuttering of the X-ray beam with on-axis viewing (OAV), shown in Fig. 1 ▸(*a*). The setup is designed to be as compact as possible to maximize flexibility and compatibility with different beamlines and sample environments such as helium or vacuum chambers, and can fit into a space of less than 170 mm × 210 mm × 140 mm. The sub-components and means of control are described in detail below.

### 2.1. Hardware   

#### 2.1.1. Shutter and attenuator   

The integrated X-ray shutter and attenuator holder are manufactured by Brandstrom Instruments (http://www.brandstrominstruments.com (part number A1084). Chosen with timing characteristics to match the experiments described below, they are pass-through shutters that have an opening time of 42 ms and a closing time of 28 ms, from activation to completion. Attenuation is achieved by means of an arm that fits into the occluding mechanism [shown in Fig. 1 ▸(*b*)] such that 10 mm × 10 mm filters of various thicknesses can be mounted without modification to the shutter. The shortest period that the shutter can open and close for is less than 100 ms (beyond that required for the experiments described below).

#### 2.1.2. On-axis viewing   

**Figure 2:**
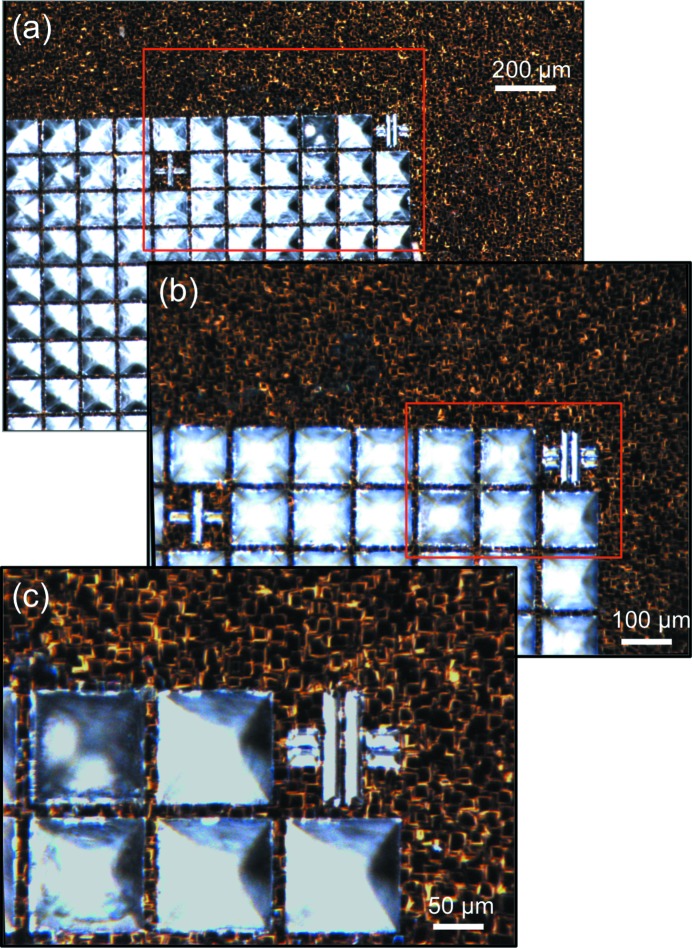
(*a*) Image quality and field of view with the different Infinitube fixed zoom lens tubes in use. The FM50 lens tube gives a field of view of 2 mm × 1.5 mm with a spatial resolution of 2 µm. (*b*) The reduced field of view achieved when using a FM100 lens tube represents an area of 2 mm × 0.7 mm, with a spatial resolution of 1.2 µm. (*c*) Further reducing the field of view with a FM200 gives an area of 0.45 mm × 0.32 mm, with a spatial resolution of 1.0 µm. The fiducial based on a # symbol is clearly observed in all figures and allows unambiguous and accurate points of reference to be established to sub-micrometer accuracy.

The OAV allows for simple alignment of objects in the X-ray beam, minimizing parallax or optical errors. The OAV also provides a high-resolution image of samples without the need for rear illumination. It comprises a Mako (http://www.alliedvision.co.uk) camera (G 125C), an Infinitube (http://www.edmundoptics.co.uk), a drilled 45° elliptical mirror (http://www.thorlabs.de), an on-axis ring light with diffuser (http://www.stemmer-imaging.co.uk) and a drilled objective lens (http://www.qioptiq.com) (28-21-10-000 10X LWD M Plan), shown in Figs. 1 ▸(*a*) and 1(*c*). The OAV configuration using the Infinitube FM100 has a very shallow depth of field in the beam direction (∼ ±10 µm), which enables the focus, or sharpness, of the image to be used as a measure of the *z*-direction in the coordinate system transformation described below. The image quality obtained is shown in Fig. 2 ▸: the nominal resolution of the viewing system is 1.2 µm (as judged using a standard USAF target). The field of view (2 mm × 0.7 mm) can be increased or decreased with a corresponding change in the spatial resolution *via* simple exchange of the Infinitube to a FM50 or FM200 (Fig. 2 ▸). The camera image can also be viewed, recorded, manipulated and processed on the same computer running the stages using the areaDetector (http://cars9.uchicago.edu/software/epics/areaDetector.html) module of EPICS (http://www.aps.anl.gov/epics/).

#### 2.1.3. Stages   

The *xyz* sample stage is a collaborative design by Diamond Light Source (DLS) and SmarAct (http://www.smaract.de), custom-built by SmarAct using three linear translation stages, each with 50 mm of travel, precise crossed roller guideways and an integrated sensor with up to 1 nm resolution. For the setup described here the resolution was decreased to 100 nm to allow an increase in the maximum velocity to 20 mm s^−1^. Motion control of the device is achieved using a DeltaTau (http://www.deltatau.co.uk) Geobrick LV-IMS-II with a standard DLS hardware configuration. The I-variable configuration file was written with *PEWIN32PRO2* software, also from DeltaTau. The stages can be controlled dynamically on a standalone Linux workstation or remotely across a network by using the EPICS Channel Access network protocol and a simple *edm* GUI displaying EPICS process variables. Sample holders are mounted to the stages using a magnetic kinematic mount allowing rapid tool-free, reproducible and accurate sample exchange.

### 2.2. Controls overview   

**Figure 3:**
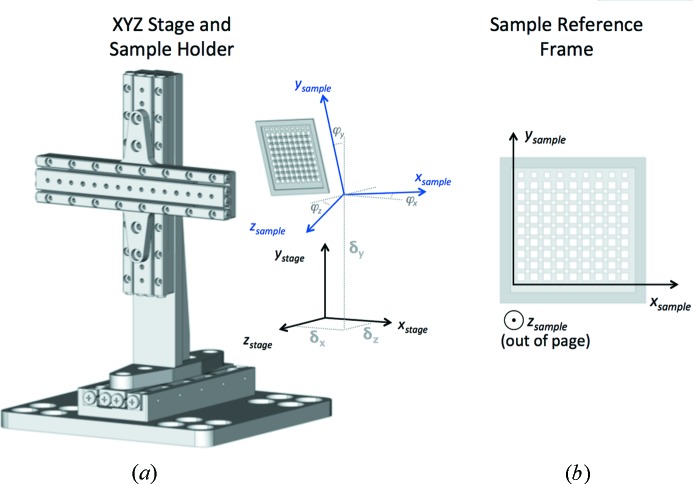
(*a*) Schematic representation of conversion of transformation between the coordinate systems of the *xyz* stages and chip sample holder. By recording the position of three reference points on the chip, the offset and angular rotation of the plane of the chip with respect to the stages is calculated. (*b*) Following the transform, points on the sample holder always have the same coordinates irrespective of how it is mounted.

**Figure 4:**
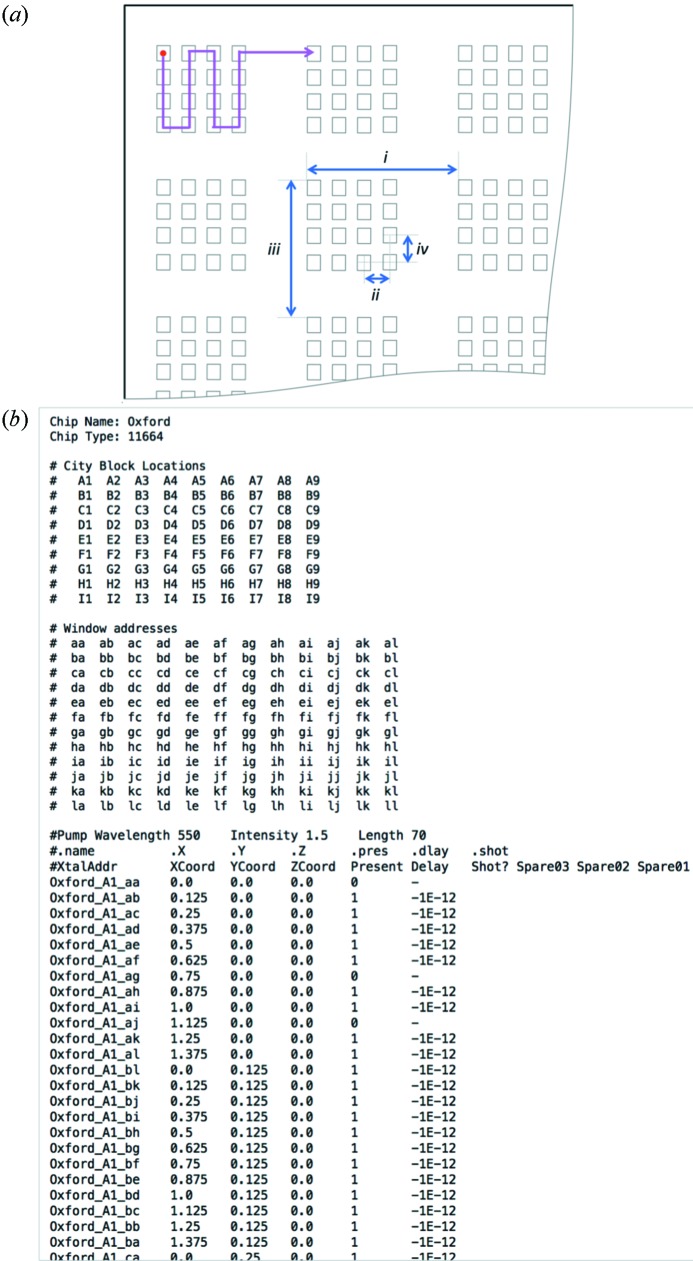
(*a*) Illustration of path followed (pink line) and the physical parameters that must be specified to generate an address file. These include the horizontal city block to city block spacing, i, the horizontal feature spacing, ii, and the vertical city block and feature spacings (iii and iv, respectively). The number of city blocks and number of features within a city block must also be specified. (*b*) Example of an automatically generated address file. The header shows the alphanumeric addressing of both city blocks and features within each block, while below the name and coordinates of apertures are listed. The additional columns allow the presence (or absence) of a sample to be flagged and experimental parameters used for each sample to be recorded.

The motion controls system converts between the coordinate system of the sample holder and that of the stages. When a sample holder is exchanged on the stages using the kinematic mounts, an accurate and quick coordinate transform is calculated. This takes account of offset from the origin, rotational misalignment and the depth of the sample holder position relative to the OAV. This is advantageous in that, irrespective of how reproducibly sample holders are mounted, a particular aperture or feature on the sample holder will always have the same coordinates; see the schematic in Fig. 3 ▸ and addressing in Fig. 4 ▸. This functionality allows the speed and accuracy of the translation stages used to be the limiting factor on throughput. Equally important (in a system for use at synchrotrons, XFELs and other large institutions) is that this greatly simplifies third-party control by allowing movement of any requested position into the X-ray beam with a single command. This ‘one command, one position’ doctrine means that prior knowledge of locations of interest on a sample holder can be easily exploited to maximize efficiency and minimize the number of blank images collected.

Locating three reference points (fiducials) on the sample holder defines a plane for the coordinate system. The fiducials can either be dedicated markers or simply recognisable features that are at the same point on each sample holder. In the example described below three corner features of the silicon nitride chip were used. Each fiducial is centered on a crosshairs on the screen and brought into the focal plane of the image: the clarity of the image and shallow depth of field provided by the OAV allows a good estimate of the translation in the *z* direction. The position of the stages at each of these fiducials is recorded to a file. The offset and three-dimensional rotation of the target can then be calculated, with the coefficients of the matrix **A**,
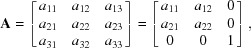
describing the transformation between the Cartesian coordinate system of the chip with respect to the Cartesian coordinate system of the stages including any scale factor and offset. For a planar sample holder this takes account of all degrees of freedom (*i.e.* offset, pitch, roll and yaw). The second, simpler, form of the matrix can be used if an offset and rotation in the *xy* plane (only) is required. The use of fiducial marks has already been proposed and exploited to characterize the orientation of sample holders; see, for example, Cohen *et al.* (2014 ▸). Here, however, the transform is implemented within the PMAC motion controller, thus ensuring full synchronization of all three axes, maximizing the speed and simplifying control commands.

**Figure 5:**
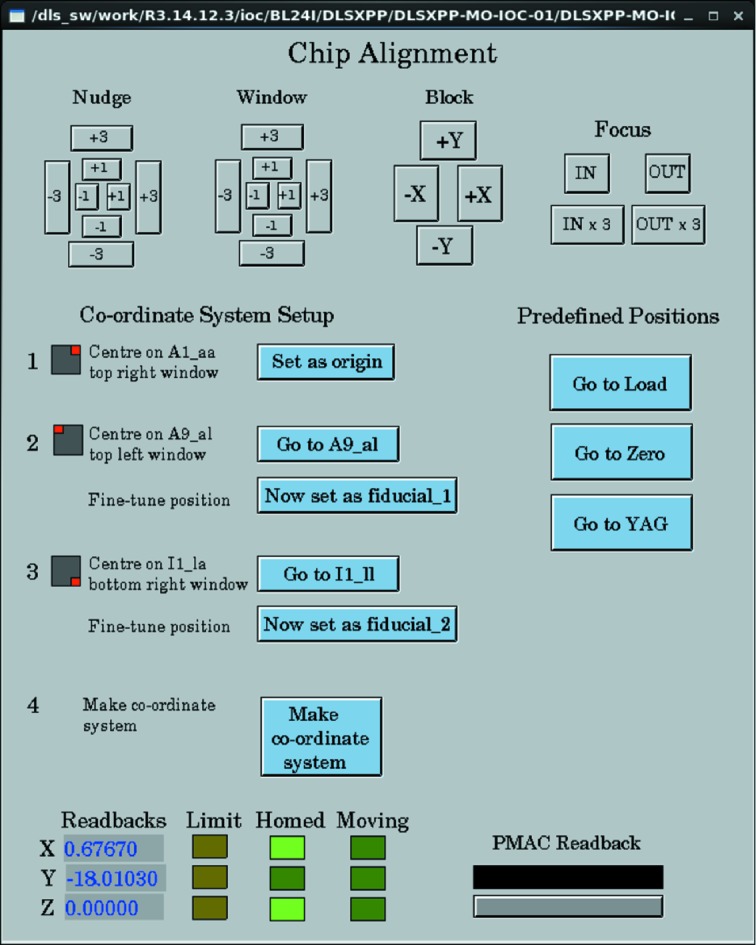
Channel Access *edm* GUI for interfacing with sample and stage. Buttons at the top provide five step sizes in the *x*,*y* direction and two for *z*. The right-hand side provides quick mounting and a laser alignment position. The left-hand side shows the procedural steps for generating the reference frame transformation matrix **A**. The bottom denotes the motor status.

A series of short scripts and an interactive GUI (Fig. 5 ▸) allow all parameters for the transform to be determined by the user in less than 60 s. Each chip axis is then defined as a combined movement of the stage axes, *i.e.*

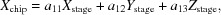


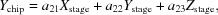


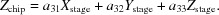
All motion of sample holders is then performed *via* requests to these virtual axes rather than movement of the individual stages.

## 3. Addressing   

So that crystals can be exposed in a controlled and repeatable manner, a simple and robust system for the automated labeling, or addressing, of each crystal was developed. This allows rapid movement between specific positions on large sample holders exploiting the coordinate system transform described above. The Boston.py software autogenerates and populates address (.addr) and shot-list (.slist) files that can be used for rastering through sample locations in a systematic way. Each sample location is labeled in the address file with a unique name/identifier together with its physical position relative to the origin and any variable experimental parameters. The addressing system uses a city grid naming convention and has similarities to the 96-well microlitre plates used in crystallization, with letters and numbers used to label rows and columns, respectively. In keeping with the city grid analogy, the top level alphanumeric are capitalized and define a city block. Addresses within any city block use lower case letters aa–zz only. The combination of chip identifier (six-letter city name), block and window locations gives each window a unique address: ‘Oxford_A1_aa’ being the upper left most window in the upper left most block of the sample holder and also the origin of the chip coordinate system (*x*, *y*, *z*) = (0, 0, 0). A more complete example of city block and feature naming is shown in Fig. 4 ▸(*b*). This naming system can provide unique addresses for more than 10^6^ locations per sample holder.

A sample section of an address file is shown in Fig. 4 ▸(*b*). Addresses are ordered within the address file such that the total distance travelled is minimized during data collection [shown schematically in Fig. 4 ▸(*a*)]. Address files can be generated by running Boston.py (this is freely available on request from the authors). Additional columns allow the presence (or absence) of a crystal at a window to be flagged so empty positions can be skipped if, for example, a sample holder is pre-screened or scored using a second setup. This has the effect of increasing both throughput and hit rate. Experimental parameters such as the desired delay of a laser pulse can also be set or recorded. The same software generates the coordinate system as well as the shot order and can also provide some basic statistics.

## 4. Performance   

**Figure 6:**
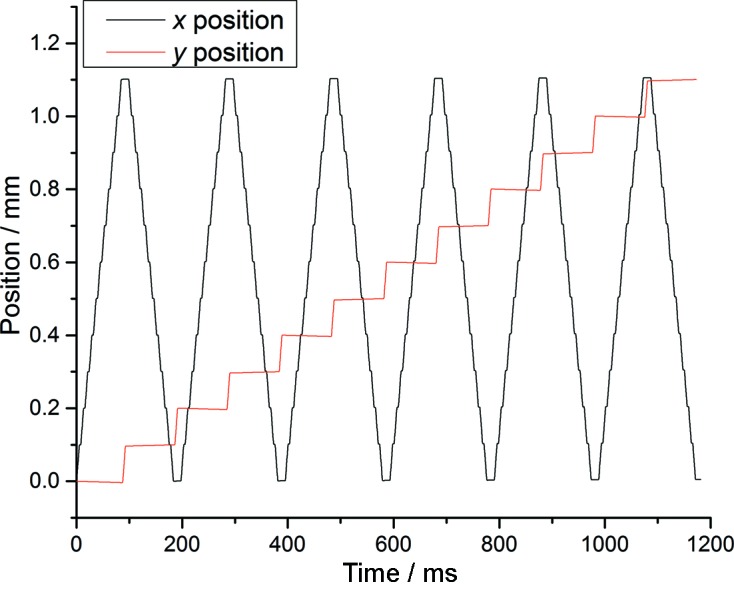
Timings and motion for a bidirectional raster scan covering a 12 × 12 grid with a pitch of 100 µm. At each position there is a dwell time of 1 ms after the encoder confirms the requested position has been reached. The rate at which sample positions are reached is 123 Hz.

The setup has been used both at Diamond Light Source and the X-ray Pump Probe (XPP) beamline (Chollet *et al.*, 2015 ▸) at the LCLS. Operation at Diamond shows the setup to be capable of positioning 100 s of samples per second with sub-micrometer accuracy. Table 1 ▸ shows measured timings for different pitches of grid, with timings for a grid of pitch 100 µm illustrated in Fig. 6 ▸. For all timings a load of 65 g was applied (Thorlabs kinematic mount plus sample mount described below) to the stages so timings represent those achievable during a diffraction experiment. If a collect–move–stop–collect protocol is applied to data collection then both the dwell time and the time overhead required for the stages to accelerate and decelerate are a significant part of the timings obtained. The stages operate in closed loop meaning there is also some time overhead while the actual position of the stages is fine-tuned before a move is completed. In order to illustrate the strategy applied and speed achieved, a video of a sample raster scan is available as part of the online supporting materials. Time overheads dominate for finely spaced grids (total overhead approximately 2.2 ms + dwell time) and result in the discrepancy between columns 3 and 4 of the table. It should be noted that gravity has a significant effect and frame rates are increased through use of a strategy where the horizontal is the ‘fast’ axis of the grid rather than the vertical. Maximum frame rates can be achieved through constant motion of the stages, and for optimal high-speed operation a hybrid ‘run and gun’ strategy of continuous motion along rows at a velocity chosen to match sample spacing to pulse rate, interspaced with motion to the next row, will be optimal. Nonetheless, it can be seen that even when a simple move–pause–move strategy is applied the pulse rate of the LCLS can be matched for arrays with a pitch of 100 µm or less. This repetition rate is not restricted to a single line of equally spaced points on, for example, a long tape but applies to a three-dimensional array. The distance of 100 µm is twice that suggested as being sufficient to completely move out of the region damaged in large cryo-cooled crystals subjected to a focused FEL beam (Hirata *et al.*, 2014 ▸).

For the experiments at XPP, photocrystallography chips (PCC) as described by Mueller *et al.* (2015 ▸) were used to mount samples. On these silicon nitride wafers, each crystal window had an opening of 20 µm and windows were spaced by 125 µm. Using the naming conventions described in §3, we defined each sub-array of 12 × 12 windows as a city block. Each PCC comprised 9 × 9 city blocks and can therefore in principal hold up to 11664 crystals, though some windows were replaced by fiducial markers reducing the maximum capacity of the chip by 6%. In order to minimize the distance travelled, a back and forth path was applied both within and between city blocks [*i.e.* alternately travelling in positive and negative directions on adjacent rows; illustrated in Fig. 4 ▸(*a*)]. During the beam time, data collection was limited to 10 Hz by the maximum frame rate of the detector used. A further limit on throughput was imposed by the desire to collect two images from each crystal, reducing the maximum window-to-window rate to 5 Hz.

Due to the high reliability of the stages, XPP control systems assumed that only window-to-window distances greater than 500 µm required a ‘done move’ from the motor controller before proceeding. Anything shorter was assumed to have arrived successfully. Including stage motion, collection method, fill types and all checks and balances, the experiment ran at 3.5 Hz over the period of any chip which worked out to average approximately 40 minutes per chip. The decreased repetition rate of 3.5 Hz was chosen to allow for additional verification steps to be added to the experimental protocol. A paper detailing data collected and methods used, including synchronization, and results obtained is in preparation. During experimental operations (∼24 h) the attenuator was triggered approximately 10^6^ times without any recorded failures either in activation or full deployment; there were two actions required at each window. For the sake of simplicity the rotary attenuator only (and not the rotary shutter) was used for these experiments. The stages performed a factor of two fewer moves, with each motion a coordinated move of three axes, with a chip window presented to the X-ray beam after every move. Experiment time included time taken for additional checks such as movement to a scintillator mounted at a predefined position on the sample holder to ensure the overlap of a crosshairs on the camera image with the X-ray and optical laser beams.

## 5. Discussion   

The hardware described provides both high-resolution visualization as well as fast and accurate positioning of sample holders for serial crystallography. The control system allows the full speed of the stages to be exploited through a coordinate system transform so any requested motion is always in the coordinate frame of the sample holder. This simplifies communication between the stages and the beamline, and means generalized address lists can be used for alignment on multiple standalone setups. Prior knowledge of crystal positions can therefore be exploited to maximize hit rates at the beamline where time is limited. Arbitrary (rather than regularly spaced) coordinates could also be generated offline by, for example, imaging crystals stuck onto thin membranes and these then used for the diffraction experiment.

The modularity and ease with which the setup can be modified including the articulate communications and simple controls mean that its use is not limited to SFX nor a particular beamline. The design could be easily adopted in areas ranging from elemental mapping, SAXS or *in situ* data collection at a synchrotron beamline. Limitations associated with a particular experiment, for example attenuator in/out timings, or spatial constraints imposed by the beamline used, can be easily circumvented by exchange or rearrangement of components. Likewise, while the current design and sealed PCC sample holders are held at room temperature, the use of modified sample holders would allow data to be collected in different ways using the hardware described here. For example, if used with a standard open flow cryostat, humidity control device or low-pressure (or helium) environment, the hardware and addressing protocols could be used in their current state for a diverse range of experiments. This modularity and flexibility, coupled with the compactness of the setup, complement the high performance of individual components to provide a flexible, portable endstation for FT-SFX.

## Supplementary Material

Video showing a sample raster scan. DOI: 10.1107/S1600577515016938/yn5006sup1.mp4

## Figures and Tables

**Table 1:** Maximum sample delivery rates for the system described *versus* the pitch of the grid used The maximum delivery rate assumes continuous motion of the stages between equally spaced features with no pause at sample positions. The measured delivery rate includes a 1 ms wait at the sample position after the encoder reports the stage to have reached the requested position.

Pitch of grid/containing the protein separation of samples (µm)	Time to move between adjacent samples (ms)	Maximum delivery rate (Hz)	Measured delivery rate with 1 ms dwell at sample location (Hz)
10	0.5	2000	257
20	1	1000	255
25	1.25	800	222
50	2.5	400	175
75	3.75	267	144
100	5	200	123
125	6.25	160	107
150	7.5	133	95
